# Notes and News

**Published:** 1889-08-10

**Authors:** 


### Notes and News.

Collected and Communicated.

At an enthusiastic meeting of members of friendly societies at Darlington, on July 25th, the following resolution was proposed by Mr. George Marshall and carried with applause: "That this public meeting of members of friendly societies approves of the scheme for the establishment of a convalescent home on the West Coast by and for the use of friendly societies, and binds itself to further its establishment by every means in its power." Dr. Boot and others spoke in favour of the scheme.

The Mayor of Warwick presided lately at the sixth annual meeting of the Hospital Saturday Committee of that town. The secretary read the balance-sheet, from which it appeared that the income from sheets, etc., was £68 11s. 11¾d.; boxes, £27 16s. 5½d.; in treasurer's hands, £1. 3s. 7d.; total, £97 12s. 0¼d. The expenditure included £8 13s. 6d. for printing, advertising, etc.; £60 was contributed to the Warneford Hospital, £1 19s. 9¼d. to the children's wards at the same institution, and £26 to the Warwick Cottage Hospital.

Falkirk Cottage Hospital has been formally opened by Mr. Brodie, of Gairdoch. The premises are situated in Thornhill-road, and consist of a cottage, which was purchased as a residence for the matron, containing parlour and kitchen, as well as an operating-room and a dispensary for the use of the medical officers on the ground-floor, with two bedrooms above. At the back of the cottage has been built the hospital proper, which consists of a brick building of two storeys, with one ward in each floor, each containing eight beds, with bathroom and other accommodation. The wards are each 34ft. by 20ft., and contain over 1,000 cubic feet of space for each patient. The buildings are heated by open fires, and ample provision is made for thorough ventilation. About £1,600 has been subscribed for the hospital, and after providing for the furnishings, there is a balance of £374 in hand.

Amongst the pictures at the Paris Salon was one of visiting day in a children's ward, which was exceedingly clever. The polished floors, white beds, and blank walls, all gave that sense of cleanliness and order which are particularly notable in French hospitals. Strange that this spotless cleanliness and marvellous polish always detract from the "home" feeling, which is specially missed by hospital children. Another cleanly custom in France is somewhat ghastly when first beheld: all the doctors and students wear large white aprons.

The popularisation of medicine goes on apace in spite of the College of Physicians. Last month Dr. Kidd gave a minute account of the symptoms and treatment of Lord Beaconsfield's last illness in the *Nineteenth Century*: an account which included much that one would hesitate to mention even at an ambulance or nursing class. It is a very ghoul-like proceeding, this gloating over the details of a death-bed; this robbing of all dignity, those on whom the king of terrors has set his seal. The cry of the great man in the future, we can well believe, will be, "Let me retire like a dog into a dark corner, and there die in peace, observed by none." Bitter indeed must be the thought that even one's latest breath may not be drawn in private, that the fierce glare of publicity follows one even through the last agony.

While so much attention is being attracted to Halifax Infirmary, the Borough Fever Hospital of that town seems to be forgotten, though it is a well-managed and cheerful institution. It was opened on May 2, 1872, for fever and smallpox cases, and 975 cases have been treated for various fevers—viz., typhoid, typhus, scarlet, and smallpox. The grounds are not large, but nicely laid out, and are pleasant to the eyes of those who dwell there, and the patients are glad to walk in them with the doctor's permission. There are four wards for typhoid cases, containing twelve beds. There are four wards for scarlet fever patients, with ten beds and cots. Each department has its own bathrooms, nurses' room, etc. The children's wants are not forgotten. There were dolls, puzzles, and toys too numerous to mention. The hospital has lately been undergoing repairs and alterations, and has been thoroughly cleaned and painted. A room is being prepared for better-class patients, an arrangement all fever hospitals ought to adopt.

The Bill for the Notification of Infectious Diseases was read a second time in the House last week. Mr. Ritchie stated the main object to be to extend the powers now exercised in 52 large towns, comprising 3,000,000 of the population, to the whole country. It was essential that notification should be given to the local authority of the existence of infectious diseases in order that they might send the officer of health and the inspector of nuisances to the place in order to take the necessary measures to prevent the spread of the disease. The Act had worked smoothly where it was in force. It was proposed that the adoption of the Act should be voluntary in the country, but compulsory in London. The diseases requiring notification were smallpox, diphtheria, erysipelas, typhoid, cholera, scarlet fever, and relapsing fever. Sir W. Foster agreed with the principle of the Bill, but thought it was going a little too far to require medical men to become informers. He would not like to see anything on the statute-book that would interfere with the confidence between the medical man and his patient. The object would be attained by making it a common duty of citizenship to give information as to infectious diseases. If the Act passed as it stood, medical men would not be called in to see infectious cases. A few other short speeches were made, and then the Bill was read.

The following vacancies are announced this week, the amount of the salary in each case being given after the name of the institution :

**Resident Medical Officers.**

Ripon Cottage Hospital. House Surgeon.—£70, board, &c.
Cornwall County Asylum. Junior Assistant Medical Officer.—£100, board, &c.
Coventry Hospital. House Surgeon.—£100, board, &c.
Salisbury Infirmary. House Surgeon.—£100, board, &c.
Chelmsford and Malden. Medical Officer of Health.—£600.

**Non-Resident Medical Officers.**

London Temperance Hospital. Registrar.—£50.
Royal Masonic Institution for Boys. Medical Officer.—£50.
Queen's Hospital, Birmingham. Physician to Out-Patients.
North Staffordshire Infirmary. Physician to Eye Department.

---

The question of establishing a Cottage Hospital at Mexborough has again been the subject of much discussion among local workmen, the result of which has been that a number of men representing various industries in the neighbourhood have formed themselves into a committee to consider the matter. They have agreed that the need is a pressing one, and that steps must be taken to forward the movement.

---

The report of the Herts Convalescent Home is out. The balance-sheet showed subscriptions received during the past year amounting to £939 6s. 6d.; church and chapel collections and entertainments, £443 4s. 1d.; donations, £41 11s. 6d.; dividends and interest, £128 16s. 8d.; patients' fees, £500 1s. 6d. The expenditure was nearly £2,000; 518 patients were admitted in the year 1888, making a total of 4,713 since the charity had its commencement. Reference was made at the meeting to the serious illness of Mrs. Philpot, the late matron, who for twelve years faithfully served the institution.

---

With reference to the two questions of leprosy and vaccination, at present much before the public, it is worth while to remember the evidence of Dr. R. Hall Bakewell before the Select Committee of 1871. Dr. Bakewell was vaccinator-general of Trinidad: "There is a strong opinion prevalent in Trinidad, and in the West Indies generally, that leprosy has been introduced to the system by vaccination. I found that medical men when they had occasion to vaccinate their own children, or those of patients in whom they were specially interested, applied to me for English lymph in order to avoid the invaccination of leprosy, notwithstanding there was an equal and probably a greater chance of English lymph being contaminated with syphilis. I had several cases of leprosy in which vaccination seemed the only means of accounting for the disease."

---

Some lectures on the training and use of the voice commence in September at Toynbee Hall; it is hoped Sir Morrell Mackenzie will be one of the lecturers. In his recent article in the *Nineteenth Century* Sir Morrell says, with regard to the "cracking" period: "I have already more than once expressed my belief that there is no reason why training, within certain limits and under strict supervision by a competent person, should not be carried on when the voice is in the transition stage of its development from childhood to adolescence. The stock argument, invariably advanced to prove the necessity of suspending the education of the voice till it has passed through the 'breaking' period is that, as the parts are undergoing active changes, they therefore require complete rest. This would equally apply to the limbs, and, in some degree, also to the brain. Yet I am not aware that it has ever been proposed to forbid growing lads from exercising their bodies, even in games involving considerable muscular violence, or to interrupt the education of the mental powers till the brain has become fully formed."

Last week the Society of Engineers visited, by invitation, the Outfall Sewage Precipitation Works at Crossness. There can be no doubt that our present sewage system is wrong; whether the Crossness one is right remains to be proved. By the precipitation method and the use of chemicals, practically all the solids will be removed, but the organic matter in solution will remain in the effluent which will go into the river, so that the evil will only be modified, and not entirely cured. The electrical treatment of sewage, which Mr. Webster showed the engineers in experimental operation, promises to remove not only the solids but 50 per cent. of the organic matter in solution. Unluckily, this method is costly.

---

A new order of the Health Board of New York makes it incumbent on a policeman to report to the nearest sanitary inspector any offensive odour that comes under their notice. The sudden zeal with which the police have assumed their new duties is not pleasing to some of the sanitary inspectors. At one o'clock one morning Inspector James Tennant was called out of bed to find a dead cat requiring his official presence at Eighty-ninth-street. He took his revenge, making a solemn official report upon the condition of the "said cat," and the recommendation that "said cat" be removed by the Health Department to the offal dock in the manner prescribed, and that its present site be disinfected. The report started on its routine through the Department that day, and by the middle of next week may be acted upon.

---

Clayton Hospital, Wakefield, expended £1,800 last year, of which £926 was contributed by working men through the Saturday Fund. The report showed that there had been an increase in the number of patients in each department compared with the previous year. There had been a considerable falling off in the amount of annual subscriptions and congregational collections, but the Hospital Saturday Fund had amounted to £82 more than last year. The president, in moving the adoption of the report and balance-sheet, referred to the distribution of recommendations, and said that more discernment ought to be used in giving them away, so that a grievance might not be caused to the medical gentlemen who gave so much of their valuable time to the Hospital. This is a remark most presidents and doctors will echo; there is nothing more annoying than careless giving away of hospital tickets.

---

New York papers are revelling in the accounts of death by electricity given before the referee, Tracy Becker, of Albany, for the purpose of getting light on the obscure questions whether an attempt to inflict death upon the murderer Kemmler with an electric current would be attended by unpleasant sensations to that individual, and whether such unpleasant sensations, if produced, would constitute a constitutional bar to the legality of this mode of execution. Mr. Gibbens, of the Board of Electrical Control described experiments he witnessed on dogs at Columbia College: the dogs did not die at once. Mr. Gibbens doubts if an electric current can be produced which will infallibly cause instantaneous death. He says: "I base my doubt upon the very different effects of electricity upon different organisms. Death by electricity, whenever it occurs, must, in my opinion, be caused by shock. If the shock is not sufficient to produce death, and the blood is merely polarised, the subject, though seemingly dead, is very likely to be kept alive and finally resuscitated by the depolarising effect of the opposite electricity with which the human system is stored. The known instances in which strong currents have failed to kill human beings and other animals also lead to the doubt whether there may not be human beings whom no current of electricity can immediately kill."

